# Computer work and self-reported variables on anthropometrics, computer usage, work ability, productivity, pain, and physical activity

**DOI:** 10.1186/1471-2474-14-226

**Published:** 2013-08-01

**Authors:** Pascal Madeleine, Steffen Vangsgaard, Johan Hviid Andersen, Hong-You Ge, Lars Arendt-Nielsen

**Affiliations:** 1 Center for Sensory-Motor Interaction (SMI), Department of Health Science and Technology, Aalborg University, Fredrik Bajers 7D, Aalborg, 9220, Denmark; 2 Danish Ramazzini Centre, Department of Occupational Medicine, Regional Hospital Herning, Gl. Landevej 61, Herning 7400, Denmark

**Keywords:** Computer use, Musculoskeletal complaints, Arm-shoulder pain, Gender, Sex

## Abstract

**Background:**

Computer users often report musculoskeletal complaints and pain in the upper extremities and the neck-shoulder region. However, recent epidemiological studies do not report a relationship between the extent of computer use and work-related musculoskeletal disorders (WMSD).

The aim of this study was to conduct an explorative analysis on short and long-term pain complaints and work-related variables in a cohort of Danish computer users.

**Methods:**

A structured web-based questionnaire including questions related to musculoskeletal pain, anthropometrics, work-related variables, work ability, productivity, health-related parameters, lifestyle variables as well as physical activity during leisure time was designed. Six hundred and ninety office workers completed the questionnaire responding to an announcement posted in a union magazine. The questionnaire outcomes, i.e., pain intensity, duration and locations as well as anthropometrics, work-related variables, work ability, productivity, and level of physical activity, were stratified by gender and correlations were obtained.

**Results:**

Women reported higher pain intensity, longer pain duration as well as more locations with pain than men (P < 0.05). In parallel, women scored poorer work ability and ability to fulfil the requirements on productivity than men (P < 0.05). Strong positive correlations were found between pain intensity and pain duration for the forearm, elbow, neck and shoulder (P < 0.001). Moderate negative correlations were seen between pain intensity and work ability/productivity (P < 0.001).

**Conclusions:**

The present results provide new key information on pain characteristics in office workers. The differences in pain characteristics, i.e., higher intensity, longer duration and more pain locations as well as poorer work ability reported by women workers relate to their higher risk of contracting WMSD. Overall, this investigation confirmed the complex interplay between anthropometrics, work ability, productivity, and pain perception among computer users.

## Background

Musculoskeletal complaints and pain located in the neck-shoulder region and upper extremities are commonly reported among computer users [1]. Musculoskeletal pain has tremendous personal and social impact leading to reduced quality of life with the possibility of loss of work and social networks [2,3]. The development of musculoskeletal pain among computer users is related to multiple factors. Individual, physical, psychosocial, and organizational factors are reported to play an important role in the development of work-related musculoskeletal disorders (WMSD) [4]. These factors interact in a complex manner in many occupations including office work [1]. Currently, the level of association between the extent of computer use and WMSD is still under debate. Two studies have found an association between mouse work and acute pain [5,6]. Concerning long-term pain complaints, some studies have suggested a causal relationship between computer work and musculoskeletal complaints in the neck-shoulder region and upper extremities [7-11] while other studies have reported moderate or no evidence for such associations in the upper extremities [1,12]. Recent studies with objective and quantitative measurements of computer use have failed to demonstrate an association between mouse and keyboard use (exposure) and prolonged pain or chronic pain [5,6,13].

Subjective assessments of symptoms in the upper extremities are most often made to study the association between computer work and musculoskeletal complaints in epidemiological studies. This has resulted in a better understanding of WMSD as well as a sound rationale for the design of interventions for the prevention of such disorders. However, self-reported exposure and musculoskeletal complaints can bias the relationship between these two entities [14]. This has also major shortcomings when handling, for instance, insurance claims [1]. Office workers often report work-related discomfort and pain even though, to date, no clear relation between computer use and neck/upper extremities disorders has been found. Women suffer approximately twice as much as men from work-related complaints in the neck and upper extremities [15-18]. Thus, studying the relationship between pain intensity, duration, and localisation in relation to physical, psychosocial, organizational, and individual factors will contribute to a better knowledge of the musculoskeletal complaints reported by computer users. This is further substantiated by the multifactorial origin of WMSD [19].

The aim of this study was to assess demographic information on short and long-term pain complaints among computer users in Denmark. The questionnaire included questions related to musculoskeletal pain, anthropometrics, work-related variables, work ability, productivity, health-related variables, and lifestyle variables (limited to the use of pain killers) as well as physical activity during leisure time. In general women are more prone to WMSD [15-17] and therefore the questionnaire outcomes were stratified by gender.

## Methods

### Subjects

An announcement concerning the present investigation was published in the magazine for HK Privat (union for office workers) reaching approx. 5000 Danish employees. Six hundred and ninety office workers completed the questionnaire which was used to screen participants as workers with or without symptoms in the neck-shoulder region and upper extremities. Another 114 workers started filling in the questionnaire but did not complete it. They were thus excluded from the subsequent analysis. Tables 1 and 2 present the anthropometric variables stratified by gender. The study was approved by the local ethics committee of The North Denmark Region (No. N-20100048).

**Table 1:** Anthropometric variables stratified by gender

	**Women**			**Men**		
	**N**	**Median**	**25-75% quartiles**	**N**	**Median**	**25-75% quartiles**
**Anthropometrics**						
Age (years)	549	48	42-54	140	52	39-58
Height (cm)	549	168	164-171	139	180	176-185
Body mass (kg)	548	68	61-79	139	84	74.5-93
Body mass index (kg/m^2^)	548	24.2	21.9-28.0	139	25.8	22.8-28.4

**Table 2:** Anthropometric, health-related and lifestyle variables stratified by gender

	**Women**		**Men**	
	**N**	**%**	**N**	**%**
**Anthropometrics**				
Overweight (Body mass index (kg/m^2^) 25-29	143	26.1	61	43.9
Obese (Body mass index (kg/m^2^) ≥30	87	15.9	15	10.8
Right handed	508	92.4	130	93.5
**Health-related variables**				
Pain caused by an accident	68	14.5	22	19.0
Cardiovascular disease	66	14.1	30	25.9
Respiratory disease	42	9.0	12	10.3
Arthritis	74	15.8	14	12.1
**Lifestyle variables**				
Regular use of pain killers (yes/no)	127/339	27.3/72.7	16/100	13.8/86.2
Regular use of pain killers due to musculoskeletal disorders (yes)	110	87.3	15	93.8

### Self-reported measures

A structured web-based questionnaire was used including questions related to anthropometrics, work-related variables, work ability, productivity, health-related variables, lifestyle variables (limited to the use of pain killers), musculoskeletal pain as well as physical activity during leisure time. The questionnaire included elements from the Standardized Nordic questionnaire for musculoskeletal disorders [20], work ability questionnaire [21], general self-efficacy [22], and the short International Physical Activity Questionnaire (IPAQ- http://www.ipaq.ki.se) [23]. The main questions of the structured self-administered questionnaire are described in more detail below:

Anthropometrics: Gender, height, body mass, body mass index, dominant side.

Musculoskeletal complaints (pain and discomfort). The pain duration, intensity, and locations were assessed. The pain duration was assessed using the number of days with complaints in a body part during the last 12 months (0 days, 1–7 days, 8–30 days, 30–90 days, more than 90 days, every day). The pain intensity was measured using a visual analogue scale (0–10 cm) anchored with 0: “no complaints/pain” and 10: “worst possible complaints/pain”. The workers scored their pain intensity within the last seven days and the last three months from the following sites: neck, right shoulder, left shoulder, right elbow, left elbow, right wrist, left wrist, and dominant forearm. The number of locations with pain (pain intensity > 0 cm) within the last seven days and the last three months was also counted. The number of painful locations was between 0 and 8 body parts. Finally, the inability to perform daily work due to complaints in the neck/dominant shoulder and dominant elbow/forearm was assessed.

Work-related variables: Position, job seniority, the number of working hours per week, percentage of time working with a computer.

Work ability compared to the person’s best lifetime and in relation to physical and mental job demands. A reduced index of work ability was calculated by summing the given score (range between 3 and 21). Values between (3–8), (9–15), (16–18), and (19–21) were defined as “poor”, “moderate”, “good”, and “excellent” work ability in agreement with [21,24].

Productivity: Ability to fulfil the company requirements on productivity. Productivity was rated on an 11-step ordinal scale anchored with 0:“the worst a worker could do” and 10: “the best a worker in the same job could do”.

Health-related variables: pain caused by an accident, suffering from e.g. cardiovascular, respiratory diseases or arthritis.

Lifestyle variables: usage of pain killers on a regular basis and, if yes, usage of pain killers due to musculoskeletal disorders. The question was limited to the use of pain killers, as it is of major relevance to the habits of the workers in relation to pain.

Physical activity during leisure time (transportation, housework or gardening, and leisure): Number of hours per week (0, <2, 2–4 and >4 hours) used to practise low (slow walking, biking not inducing huffing/sweating), moderate (walking, biking inducing huffing/sweating), and vigorous exercise (competitive sports). The physical activity reported by the workers was converted into metabolic equivalent task (MET) × min × week^-1^ according to the guidelines for data processing of the IPAQ. Further, workers were classified into one of three categories noted as low, moderate, and high based on the reported level of activity. Workers in low activity performed < 600 MET × min × week^-1^. Workers in moderate activity achieved between 600 and 3000 MET × min × week^-1^ while workers achieving >3000 MET × min × week^-1^ were categorized as high activity in line with the recommendations set by the American College of Sports Medicine [25].

The tasks carried out by the workers were characterized as office work (administrative tasks) without special physical loads. Workers reporting a pain intensity >0 cm on the visual analogue scale in neck, shoulders, and arms were considered having musculoskeletal pain. Workers reporting pain intensity = 0 were considered pain free. Therefore, an explorative analysis including workers with and without musculoskeletal pain was performed. The investigation enabled a detailed study of a population of computer users gaining information on the relations between anthropometrics, work-related variables, work ability, productivity, musculoskeletal pain, health-related variables, lifestyle variables, and physical activity during leisure time.

### Data analysis

Median [25-75% quartiles] values are reported in text and tables. Mann–Whitney U-test was used to compare the effects of gender on the following independent variables: age, BMI, number of hours/week working with a computer, work ability, ability to fulfil the company requirements on productivity, the frequency of pain in forearm-elbow-neck-shoulder, the pain intensity in the forearm-elbow-neck-shoulder over the last seven days and three months, the number of locations with pain, and the distribution of the level of physical activity. Spearman’s correlation coefficient was computed for both men and women to delineate the relationship among these independent variables. P < 0.05 was considered significant.

## Results

### Gender differences

**Figure 1:**
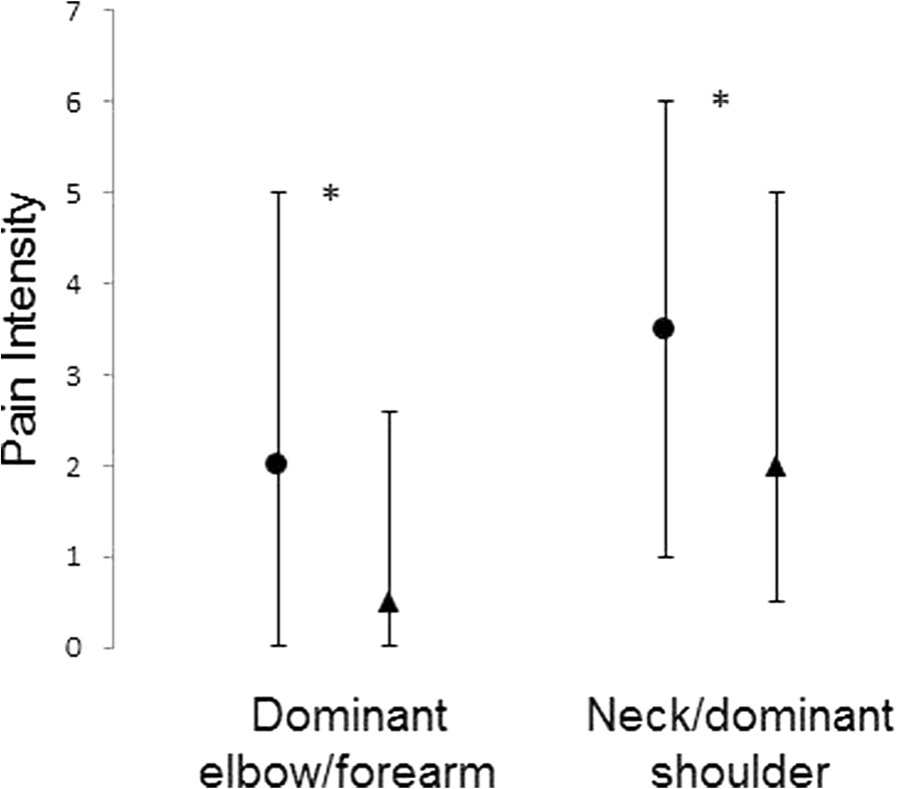
**Pain intensity for women and men.** Median and 25-75% quartiles pain intensity within the last three months in the dominant elbow/forearm and neck/dominant shoulder for women (●) and men (▲). * P < 0.05.

Tables 1 and 2 report anthropometrics, health-related and life style variables stratified by gender. Women were younger (P = 0.03) and had lower BMI (P = 0.02) than men. Table 3 shows work-related, work ability, and productivity variables stratified by gender. The job seniority was 18 [10–26] years for the women (N = 529) and 15 [10–25] years for the men (N = 125). The number of working hours/week was 37 [35–37] hours for the women (N = 529) and 37 [37–40] hours for the men (N = 125). Women reported poorer work ability than men (P = 0.01). Women (9 [8–9], N = 499) reported poorer ability to fulfil the company requirements on productivity than men (9 [8–9], N = 119, P = 0.02). Tables 4, 5 and 6 report pain frequency, pain intensity in the neck/dominant shoulder and dominant elbow/forearm, and the number of locations with pain stratified by gender. Women had higher frequency of pain in the dominant forearm-elbow-neck (P = 0.027, P = 0.025 and P = 0.001, respectively), higher pain intensity within the last seven days in the dominant forearm-neck (P = 0.037 and P = 0.001, respectively) and within the last three months in the forearm-elbow-neck (P = 0.002, P = 0.007, and P = 0.005, respectively) as well as more locations with complaints (P = 0.03) than men. Figure 1 shows the pain intensity within the last three months in the dominant elbow/forearm and the neck/dominant shoulder for women and men. Further, both women and men reported inability to perform daily work due to complaints in the neck/dominant shoulder (104 (21.5%) and 20 (16.9%), respectively) and dominant elbow/forearm (75 (15.9%) and 8 (6.9%), respectively). Table 7 reports the level of physical activity during leisure time stratified by gender. The level of physical activity was 1680 [1272–2160] MET × min × week^-1^ for the women (N = 465) and 1752 [1272–2160] MET × min × week^-1^ for the men (N =116).

**Table 3:** Work-related, work ability, and productivity variables stratified by gender

	**Women**		**Men**	
	**N**	**%**	**N**	**%**
**Work-related variables**				
Percentage of time working with computer				
Almost always	338	63.8	55	44.0
¾	133	25.1	50	40.0
½	40	7.5	15	12.0
¼	12	2.2	3	2.4
Seldom	4	0.8	1	0.8
Never	3	0.6	1	0.8
**Work ability** (reduced index)				
Poor	11	2.2	1	0.8
Moderate	92	18.0	14	11.6
Good	168	32.9	33	27.3
Excellent	239	46.9	73	60.3
**Productivity**				
Diseases affecting work (yes)	146	26.5	24	17.1

**Table 4:** Frequency of musculoskeletal complaint variables stratified by gender

	**Women**		**Men**	
	**N**	**%**	**N**	**%**
**Musculoskeletal pain**				
Number of days with neck/dominant shoulder complaints in the last 12 months*				
0	51	10.4	19	15.7
1-7	86	17.5	31	25.6
8-30	93	18.9	21	17.4
31-90	59	12.0	11	9.1
>90	106	21.5	20	16.5
Every day	97	19.7	19	15.7
Number of days with dominant elbow/forearm complaints in the last 12 months*				
0	159	33.3	49	42.2
1-7	70	14.7	18	15.5
8-30	69	14.5	16	13.8
31-90	57	11.9	9	7.8
>90	64	13.4	17	14.7
Every day	58	12.2	7	6.0

* Highest number of days with complaints was taken in consideration.

**Table 5:** Intensity of musculoskeletal complaints variables stratified by gender

	**Women**			**Men**		
	**N**	**Median**	**25-75% quartiles**	**N**	**Median**	**25-75% quartiles**
**Pain intensity within the last 7 days**						
Forearm	473	0	0-3	116	0	0-1.3
Elbow	478	0	0-4	116	0	0-2.3
Shoulder	484	1	0-6	118	1	0-4.8
Neck	490	2	0-5	119	0	0-4
**Pain intensity within the last 3 months**						
Forearm	473	1	0-5	116	0	0-3
Elbow	478	1	0-6	116	0	0-3
Shoulder	484	3	0-7	118	2	0-6
Neck	491	3	1-6	119	2	0-5

**Table 6:** Number of locations with pain (pain intensity > 0) ranging from 0 to 8 body parts within the last seven days and the last three months stratified by gender

	**Women**				**Men**			
	**N**	**%**	**N**	**%**	**N**	**%**	**N**	**%**
**Body part with pain**	**Within the last 7 days**		**Within the last 3 months**		**Within the last 7 days**		**Within the last 3 months**	
W	238	50.1	279	58.7	43	37.1	56	48.3
NDW	178	37.5	212	44.6	16	13.8	20	17.2
E	211	44.1	255	53.3	45	38.8	51	44.0
NDE	109	22.8	132	27.6	15	12.9	17	14.7
F	210	44.4	261	55.2	43	37.1	52	44.8
S	274	56.5	337	69.5	62	53.4	78	67.2
NDS	178	36.7	212	43.7	30	25.9	37	31.9
N	310	63.1	397	80.9	54	46.6	85	73.3
W + E	158	33.3	204	42.9	26	22.4	34	29.3
E + S	172	36.4	224	46.9	33	28.4	39	33.6
S + N	234	48.2	306	63.1	45	38.8	64	55.2
E + S + N	153	32.0	206	43.1	28	24.1	35	30.2
W + E + S + N	125	26.3	177	37.3	17	14.7	23	19.8
W + E + F + S + N	113	23.9	156	33.0	17	14.7	22	19.0
W + NDW + E + F + S + N	87	18.4	105	22.2	6	5.2	9	7.8
W + E + NDE + F + S + N	67	14.2	84	17.8	7	6.0	8	6.9
W + E + F + S + NDS + N	87	18.4	105	22.2	10	8.6	12	10.3
W + NDW + E + NDE + F + S + N	66	14.2	76	16.1	6	5.2	7	6.0
W + E + NDE + F + S + NDS + N	66	14.2	76	16.1	6	5.2	7	6.0
W + NDW + E + NDE + F + S + NDS + N	66	14.2	76	16.1	6	5.2	7	6.0

Locations: dominant wrist (W), elbow (E), forearm (F), shoulder (S), neck (N) and non-dominant wrist (NDW), elbow (NDE), shoulder (NDS).

**Table 7:** Level of physical activity stratified by gender

	**Women**		**Men**	
	**N**	**%**	**N**	**%**
**Physical activity during leisure time**				
Low	24	5.2	12	10.4
Moderate	408	87.6	94	81.0
High	33	7.2	10	8.6

Levels of physical activity: Low: below 600 MET × min × week^-1^, moderate: between 600 and 3000 MET × min × week^-1^ and high: above 3000 MET × min × week^-1^. MET: metabolic equivalent task.

### Correlations between arm-neck-shoulder pain and anthropometrics / work-related variables / work ability / productivity / physical activity

**Figure 2:**
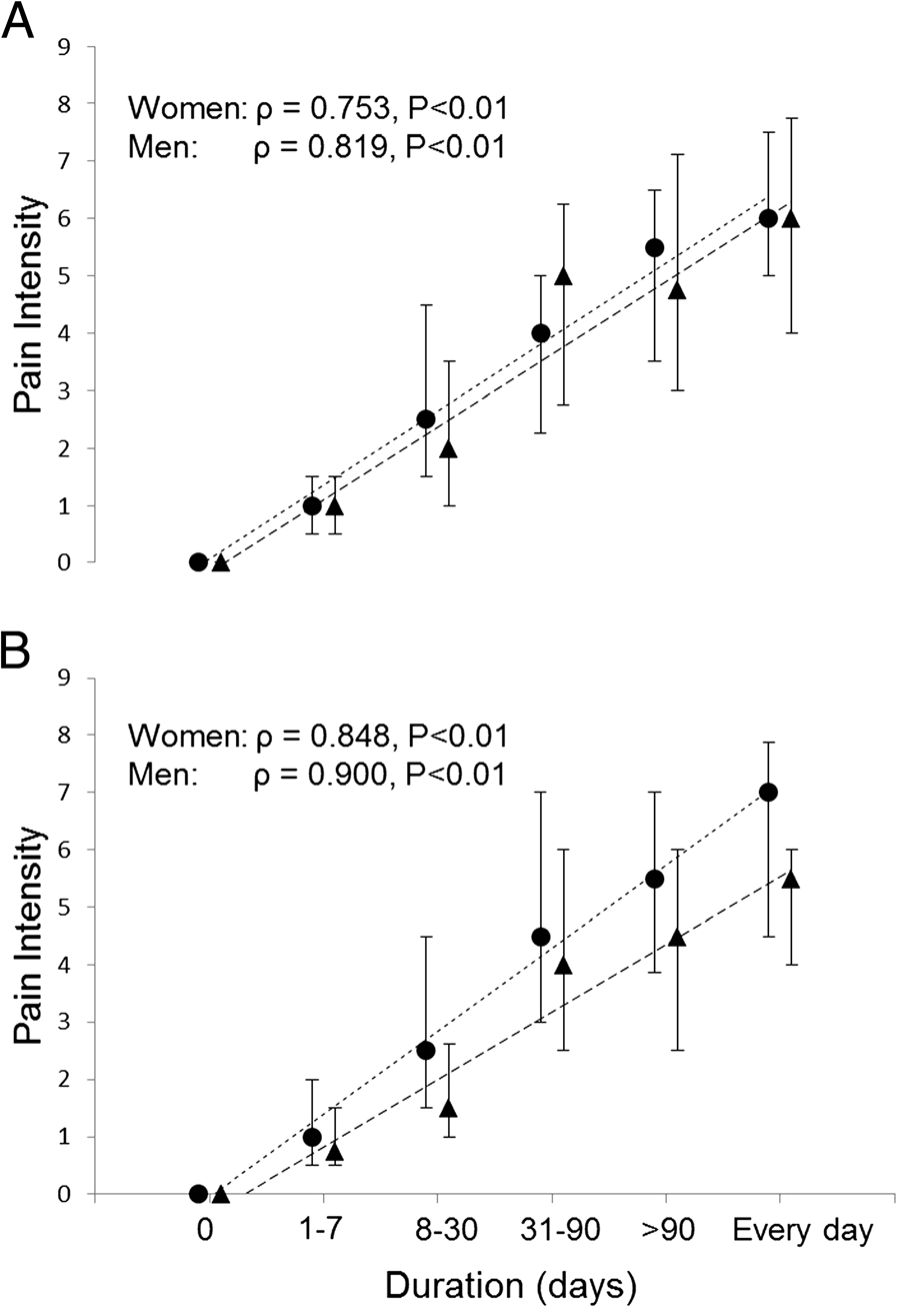
**Pain intensity and pain duration relationship for women and men.** Median and 25-75% quartiles pain intensity within the last three months and pain duration within the last 12 months (highest number of days with complaints) for women (●) and men (▲) in **(a)** the neck/dominant shoulder and **(b)** the dominant elbow/forearm. ρ: Spearman’s correlation coefficient and P: level of significance.

Significant positive correlations were found between pain intensity within the last seven days and pain duration for women in the forearm, elbow, neck, and shoulder (ρ = 0.83, P <0.001, ρ = 0.83, P < 0.001, ρ = 0.75, P < 0.001, and ρ = 0.81, P < 0.001, respectively) and for men in the forearm, elbow, neck, and shoulder (ρ = 0.81, P < 0.001, ρ = 0.86, P < 0.001, ρ = 0.71, P < 0.001, and ρ = 0.80, P < 0.001, respectively). Similarly, significant positive correlations were found between pain intensity within the last three months and pain duration for women in the forearm, elbow, neck, and shoulder (ρ = 0.91, P < 0.001, ρ = 0.90, P < 0.001, ρ = 0.75, P < 0.001, and ρ = 0.87, P < 0.001, respectively) and for men in the forearm, elbow, neck, and shoulder (ρ = 0.89, P < 0.001, ρ = 0.93, P < 0.001, ρ = 0.81, P < 0.001, and ρ = 0.91, P < 0.001, respectively). Figure 2 shows the relationship between pain intensity within the last three months and pain duration within the last 12 months (highest number of days with complaints) for women and men in the neck/dominant shoulder and the dominant elbow/forearm.

A significant positive correlation was also found between the overall pain intensity (sum of the eight locations) within the last three months and BMI for men (ρ = 0.20, P = 0.02). Further, significant negative correlations were found between the overall pain intensity within the last three months and work ability or productivity for women (ρ = −0.47, P < 0.001 and ρ = - 0.32, P < 0.001, respectively) and for men (ρ = −0.40, P < 0.001 and ρ = 0.-42, P < 0.001, respectively). No other significant correlations were found.

## Discussion

The present study extends the knowledge on pain and gender providing data concerning the relationship between computer usage, work ability, productivity, physical activity, and self-reported pain. The results provide novel findings on musculoskeletal complaints and gender among office workers. Positive correlations were found between 1) pain intensity and pain duration and 2) pain intensity and number of pain locations.

Musculoskeletal disorders constitute a societal and economical problem [3]. Complaints of pain located in the neck-shoulder region and upper extremities are commonly reported by computer users. The prevalence of complaints in the neck and upper extremities is reported to be approximately twice as high among women as compared with men [15-17]. The present findings concerning the frequency of pain and the number of locations with pain corroborated the higher WMSD prevalence reported in women. The distribution of the self-reported complaints from the dominant arm-forearm and the neck-shoulder differed among gender, with women having more days with symptoms over the last 12 months (Table 4). In parallel, women reported higher pain intensity within the last 7 days or 3 months over the dominant forearm-elbow-neck-shoulder than men (Table 5). WMSD are also characterised by a spreading of pain to larger body areas [26,27]. In agreement with this latter statement, the women who responded to our questionnaire also reported more locations with complaints than men (Table 6). This is of importance as an increase in the number of painful regions is found to be associated with a poorer prognosis [28]. The reported differences in musculoskeletal pain frequency, intensity, and spreading are well in line with the known gender differences in pain perception [29-32]. Several biological and biosocial mechanisms such as gonadal hormones, genetic factors, and multiple psychological factors have been proposed to account for these gender-related differences. Psychosocial factors such as gender role expectancies, beliefs regarding the ability to control and tolerate pain as well as anxiety have been suggested [31,32]. Therefore, the lower scores on work ability and lower ability to fulfil the requirements on productivity reported by the female computer users compared with males have probably contributed negatively to the overall pain perception in agreement with previous findings [33]. Negative mood states relative to work or family, such as anxiety and depression, are also suggested to be potent contributors to gender differences in pain perception [31,32]. Biological factors encompass for instance different sex-related hormonal influences, resting blood pressure, genetic influences, mechano-sensitivity, and descending inhibitory pain control [29-31]. Both age and BMI differed among groups in the present study. However, these differences further substantiate the reported differences in pain and work ability. Age and BMI are indeed known risk factors for musculoskeletal pain [34-36]. Based on this point and on the study sample size, the analysis was not adjusted for age, BMI or other socio-economic variables. From a clinical point of view the reported gender differences in pain characteristics (frequency, intensity and spreading) and work ability among computer users provide further arguments for the larger extent of prolonged or chronic WMSD in the upper extremities reported in women [15-17].

The present study also investigated the correlations between pain located in the arms or neck-shoulder region and anthropometrics, work-related variables, work ability, productivity, and physical activity. The time spent working with a computer has been reported as a predictor for future musculoskeletal symptoms in prospective studies investigating complaints from the hand and wrist [37,38]. The time spent using a computer mouse is also found to be related to forearm and shoulder complaints [39]. The number of hours of daily or weekly computer work is positively associated with more arm-hand than neck-shoulder complaints [10]. At baseline the NUDATA study has shown that working with a computer mouse for more than 15–20 hours/week is associated with a risk of tension neck syndrome [40]. Contrary to these studies, this study did not find any significant correlation between the number of hours reported working with a computer and the pain intensity reported from the dominant forearm-elbow and neck-shoulder as well as the number of locations with pain. This is actually in line with recent prospective studies reporting that the duration of mouse and keyboard use is unrelated to prolonged or chronic musculoskeletal pain conditions [5,6,13]. The lack of relationship between pain and duration of computer use is further supported by the fact that severe pain does not have a better prognosis if mouse and computer work is carried out for shorter duration [28]. Interestingly, the neck-shoulder region has been found less susceptible to exposure in connection with computer usage than the hand-arm region [8,10]. This is in agreement with the relationship found between the time of mouse usage and acute distal arm pain [6]. However, a possible link between complaints in the neck-shoulder and distal arm pain has been reported during mouse usage [41]. A case–control study showed a small association between seeking care because of neck or shoulder disorders and computer work for more than 4 hours/day among women [42]. This is in line with the slightly higher pain prevalence reported among women [6]. Concerning sickness absence computer professionals and technicians have been found to have a low risk of sickness absence [43] and computer use did not predict future long-term sickness absence [44]. The present results and the existing literature confirm that musculoskeletal pain in computer users probably results from complex relationships between individuals, societal and work-related aspects.

The current study revealed positive strong correlations between pain intensity (reported for the last seven days and three months) and pain duration for the forearm, elbow, neck, and shoulder. These correlations show that the higher the pain intensity the longer it will last. This result suggests that both ongoing pain intensity and pain duration can play an important role in the chronification of WMSD or the recurrence of symptoms. Chronic musculoskeletal pain is often associated with spatial pain propagation in agreement with the number of painful locations reported in the current study [26,45]. Along with the chronification, more and more sensory abnormalities occur; on-going peripheral nociception may aggravate peripheral and central sensitization. An imbalance in pain modulation is reported to lead to a long-lasting pain process [46]. However, further studies are needed to examine the peripheral and central neuro-modulation mechanisms among computer users suffering from pain. Thus, the relationship between pain intensity and pain duration can have important implications in terms of generalized hypersensitivity to pain reported in clinical conditions like musculoskeletal disorders [26] and osteoarthritis [47]. This study also found a moderate negative correlation between the overall pain intensity within the last three months and work ability or productivity underlining the interplay between pain intensity and work ability or productivity. Such a relationship is important as low work ability is a good predictor of long-term sickness absence [48].

Physical activity has been shown to counteract the development of WMSD to some extent [49]. As such, physical activity has also been shown to lead to a reduction in the pain intensity [50]. Moreover, physical activity can result in lower values for BMI, body fat percentage and blood pressure as well as better work performance [51]. Recently, a relationship between vigorous-intensity physical activity and lower neck pain has been reported suggesting a possible preventive role of physical activity on musculoskeletal pain [23]. Contrary to these studies this study did not find any significant correlation neither between the level of physical activity and the overall pain intensity nor between the level of physical activity and the number of locations with pain among computer users. This can most likely be explained by the skewed distribution of the physical activity during leisure time among the computer workers (Table 7). According to the IPAQ classification, approximately 88% of the workers belonged to the physically active category in relation to the recommendation of the American College of Sports Medicine [25]. It is likely that some of these workers overestimated their level of physical activity since 40% of all adults in Denmark do not fulfil the recommendation for physical activity [23,52]. Another explanation is related to the fact that the IPAQ does not enable detection of relatively small changes like 1 hour/week in physical activity [23]. Further studies investigating the level of physical activity using objective measures and pain perception are warranted.

Finally, it is acknowledged that the present study has limitations. Firstly, all participants came from a selected group of computer workers in Denmark that may not be representative of all computer users. On the other hand, this could also be considered a strength as the investigated group was rather homogenous in terms of exposure. The invitation to participate reached approximately 5000 office workers from the union HK Privat (union for office workers). The age of the female workers filling in the questionnaire was similar to the age of the rest of employees (47 years) while the male participants were younger (4 years on average). The gender distribution differed as the woman/man ratio was 4 for the participants compared with a ratio of 2.7 for the entire HK Privat. As reported by Strazdins and Bammer [32], a self-selection bias may have occurred. It is possible that the workers completing the questionnaire were more concerned with their health. No knowledge about the non-respondents exists and no further elaboration on the self-selection bias can be made. Further studies elaborating this issue in relation to gender are needed. Only self-reported data about anthropometrics, work-related variables, work ability, productivity, musculoskeletal pain, health-related variables, lifestyle variables, and physical activity during leisure time were collected. This is a serious limitation inherent to all questionnaire-based studies (e.g. overestimation of the outcome) as for instance self-reporting of duration of computer work is inaccurate compared with objective measurements. However, it has been shown that self-reported computer usage reflects objective usage, especially with higher levels of usage as in the present cohort [14]. Further, the collection of self-reported data concerning both exposure and its effects in one single questionnaire may have introduced a bias in the responses given by the workers [14]. A way to address this issue is to add or replace the questionnaire with recordings of computer use and clinical examinations. In this study the frequency, intensity, and locations of symptoms were collected to obtain a broad pattern of symptoms in line with [16]. However, the solely subjective assessments of pain do not necessarily relate to possible pain mechanisms. More studies elaborating on pain mechanisms using quantitative sensory testing among computer users [53] are needed to obtain a more profound understanding of the WMSD [1]. Issues related to ergonomics, i.e., working posture and motor control were not addressed even though these are known to play a role in the gender differences found in WMSD [18,54-56]. Thus, confounding factors related to uncontrolled ergonomic, psychosocial, and personal factors cannot be excluded.

The participation rate was low (approx. 14%) and this may have induced bias in the self-reported outcomes and self-reported computer usage in agreement with [6]. The relatively small sample size (especially for men) and the data distribution for physical activity may have affected the power to detect more significant differences and correlations. Despite these limitations, this study is to our knowledge the first investigation focusing on gender aspects in relation to pain perception, work ability, productivity, and physical activity among computer users.

## Conclusions

On the basis of a questionnaire, musculoskeletal pain, anthropometrics, work-related variables, work ability, productivity, health-related variables, lifestyle variables as well as physical activity were analysed among 690 Danish computer users. Differences in pain perception, i.e., higher intensity, longer duration and more pain locations as well as poorer work ability were reported by women compared with men. These findings relate to the higher risk of contracting WMSD found in women. Further, the present analyses confirmed the complex interplay between anthropometrics, work ability, productivity, and pain perception, i.e., pain intensity, duration and locations among computer users.

## Consent

Written informed consent was obtained from the participants for the publication of this report.

## Abbreviations

BMI: Body mass index; IPAQ: International physical activity questionnaire; MET: Metabolic equivalent task; WMSD: Work-related musculoskeletal disorders.

## Competing interests

The authors declare no competing interests.

## Authors’ contributions

LAN, PM and GHY conceived the research idea. LAN, PM, JHA and GHY discussed and wrote the initial protocol as well as the design of the study. PM was responsible for drafting the paper. LAN was responsible for the application to the ethical committee. PM, SV, GHY, LAN and JHA discussed the analyses. SV and PM performed the measurements and analysed the data. All authors have read and commented on the draft version and approved the final version of the manuscript.

## Pre-publication history

The pre-publication history for this paper can be accessed here:

http://www.biomedcentral.com/1471-2474/14/226/prepub
